# Characterization of Phenolic and Essential Oil Constituents of *Satureja boissieri* Hausskn. ex Boiss and Evaluation of Antioxidant Potential

**DOI:** 10.3390/molecules31101710

**Published:** 2026-05-18

**Authors:** Sema Çarıkçı, Tuncay Dirmenci, Ilhami Gulcin, Ahmet C. Goren

**Affiliations:** 1 Vocational School, Izmir Demokrasi University, 35140 Izmir, Türkiye; 2 The Sustainable Environmental Studies Application and Research Centre, Izmir Demokrasi University, 35140 Izmir, Türkiye; 3 Department of Biology Education, Necatibey Faculty of Education, Balikesir University, 10145 Balikesir, Türkiye; dirmenci@balikesir.edu.tr; 4 Chemistry Department, Faculty of Science, Atatürk University, 25240 Erzurum, Türkiye; igulcin@atauni.edu.tr; 5 Rectorate of Agri Ibrahim Cecen University, 04100 Agrı, Türkiye; 6 Department of Chemistry, Faculty of Basic Sciences, Gebze Technical University, 41400 Kocaeli, Türkiye; 7 Troyasil HPLC Column Technologies, Doruk Analitik, Mehmet Akif Mah, Yumurcak Sok, No. 43, Istanbul 34744, Türkiye

**Keywords:** *Satureja boissieri* Hausskn. ex Boiss, secondary metabolite, LC-HRMS, antioxidant activity, cholinergic activity, syringic acid, carvacrol

## Abstract

This study investigated the chemical composition of the essential oil (EO) and the phenolic profiles of methanol extracts from *Satureja boissieri* Hausskn. ex Boiss. EO analysis by Gas Chromatography–Mass Spectrometry (GC-MS) identified carvacrol (45.2%), cymene (26.0%) and γ-terpinene (17.5%) as the primary constituents. Phenolic profiles were quantified via Liquid Chromatography–High-Resolution Mass Spectrometry (LC-HRMS), revealing syringic acid (56,647.96 mg/kg extract), rosmarinic acid (47,777.98 mg/kg extract) and hesperidin (6353.49 mg/kg extract) as major components in the extract. The antioxidant potential was evaluated through three distinct radical scavenging assays (DPPH, ABTS and DMPD) and the determination of ferric (Fe^3+^) and cupric (Cu^2+^) reducing capacities. Notably, *S. boissieri* exhibited potent antioxidant activity, with IC_50_ values of 11.74 µg/mL for DPPH and 9.90 µg/mL for ABTS radical scavenging, demonstrating performance comparable to standard antioxidants such as α-tocopherol (11.31 and 8.37 µg/mL, respectively). Furthermore, the in vitro inhibitory activities against acetylcholinesterase (AChE) and butyrylcholinesterase (BChE) enzymes were evaluated. While both extracts exhibited similar and significant AChE inhibition (35.3% and 32.4%, respectively), the essential oil was notably more potent against BChE than the methanol extract. These findings suggest that *S. boissieri* is a significant source of bioactive compounds with promising antioxidant and neuroprotective potential for pharmaceutical applications.

## 1. Introduction

The significant genus *Satureja* L., characterized by its potent aromatic properties. It is represented by 45 species (53 taxa) worldwide and is a prominent member of the Lamiaceae family. This family encompasses numerous taxa rich in medicinal and aromatic attributes, with specialized species distributed throughout the Mediterranean. Türkiye is notably rich in *Satureja* diversity, providing a habitat for 18 taxa, including six endemics [1,2,3,4,5,6]. The etymology of the genus remains a subject of debate, reflecting its intertwined medicinal and mythological history. According to Carl Linnaeus, the name originates from the Latin root “satura” (meaning “satiated”), which is reference to the plant’s historically esteemed digestive properties [7]. Conversely, an alternative etymological lineage connects the genus to the “satyr” of Roman and Greek mythology. Known as the “herb of the satyr,” it was celebrated in antiquity for its robust aroma and supposed aphrodisiac effects. Its reputation was so strong that its cultivation was famously prohibited in certain medieval monasteries [8].

Known worldwide as “savory” and in Anatolia as “Sivri Kekik” and “Taş Kekiği,” these plants have been staples in local cuisine and folk medicine since ancient times [7]. Traditionally used to treat gastrointestinal distress and myalgia, *Satureja* species are often brewed into therapeutic teas for respiratory relief. Modern scientific literature confirms their diverse pharmacological profile, including antimicrobial, anti-inflammatory, and diuretic properties [8,9,10,11]. Although it has been reported that nearly all *Satureja* species are used by local communities, certain taxa are of greater commercial significance. Among those with high market value are *S. hortensis* L., *S. cuneifolia* Ten., *S. thymbra* L., and *S. boissieri* Hausskn. ex Boiss [12,13].

*Satureja boissieri* is an aromatic perennial herb and an endemic subshrub native to eastern Turkey. Characterized by a dense arrangement of white flowers, it thrives primarily in arid climates [5]. Locally used as a culinary herb (“kekik/Catri”) and traditional remedy, its chemical profile is dominated by an essential oil (EO) rich in carvacrol—typically ranging from 40–45% in Turkish material to nearly 70% in Iranian populations—alongside notable amounts of γ-terpinene and p-cymene [14,15,16,17]. Extracts from its leaves contain high levels of phenolic compounds, particularly hesperidin and rosmarinic acid, which confer robust in vitro antioxidant capacity. These results are comparable to standard antioxidants such as ascorbic acid, BHA, and BHT [18]. Furthermore, these phenolics and terpenoids underline measurable antibacterial and antifungal activities against a spectrum of Gram-positive/Gram-negative bacteria and filamentous fungi [15,18]. Isolated components, such as p-cymene and thymol, have also demonstrated marked cytotoxic effects against HeLa cervical cancer cells in vitro, suggesting potential anticancer relevance, albeit currently limited to cell culture studies [19]. Overall, *S. boissieri* emerges as a chemically rich species with promising antioxidant, antimicrobial, and cytotoxic properties. Consequently, a comprehensive phytochemical characterization and a detailed evaluation of its biological efficacy are of paramount importance to elucidate its full therapeutic potential and industrial applicability [9,10,11,14].

Natural products remain indispensable reservoirs for modern drug discovery, with a significant proportion of pharmaceuticals being derived from or inspired by these complex natural scaffolds [20,21]. The therapeutic potential of *Satureja* is largely attributed to its high content of phenolic monoterpenes, which play a critical role in mitigating oxidative stress [22,23,24]. This is particularly vital as excess reactive oxygen species (ROS) and nitrogen species are recognized as primary catalysts of cellular dysfunction in pathologies such as diabetes, cancer, and neurodegenerative or cardiovascular diseases [25]. Beyond traditional uses, the incorporation of standardized plant extracts into functional food supplements has gained significant momentum. Therefore, there is an increasing trend toward ethnobotanical and phytochemical screening to identify novel, antioxidant-rich taxa. Systematic investigations into *Satureja* taxa are essential not only to validate their traditional use but also to identify novel lead compounds for multi-target therapies against neurodegenerative disorders [8].

Within this framework, the biological activities of both methanol extracts and EOs derived from the aerial portions of *S. boissieri* were systematically investigated. Unlike prior studies that focus on general extracts, this research provides a comprehensive quantification of phenolic compounds—natural antioxidant molecules commonly found in plants—using liquid chromatography–high resolution mass spectrometry (LC-HRMS), an advanced technique for separating and identifying chemicals. Each compound is supported by rigorous, complete analytical validation, ensuring unprecedented precision in the phytochemical profiling of *Satureja boissieri*. The chemical profile of the EO was identified by Gas Chromatography–Mass Spectrometry (GC-MS). Furthermore, both preparations were evaluated for their potential neuroprotective properties using inhibitory assays against acetylcholinesterase (AChE) and butyrylcholinesterase (BChE) enzymes. The antioxidant capacity was also rigorously assessed using a multi-analytical approach, encompassing three radical scavenging assays (DPPH**^•^**, DMPD**^•^**^+^, and ABTS**^•^**^+^) alongside two metal-reducing power evaluations (FRAP and CUPRAC).

## 2. Results and Discussions

### 2.1. Essential Oil

The chemical profile of the essential oil from *Satureja boissieri* was characterized by the identification of 46 individual components, representing 99.7% of the total oil (Table 1). The analysis reveals that oil is predominantly composed of monoterpene hydrocarbons (49.8%) and phenolics (46.5%), while sesquiterpenes constitute only a minor fraction (1.4%). The most abundant single component identified was carvacrol (45.2%), followed by p-cymene (26.0%) and γ-terpinene (17.5%). These findings are largely in line with the existing literature on Turkish *Satureja* species, which reports carvacrol (21.3–44.8%), p-cymene (23.2–35.5%), γ-terpinene (6.5–26.4%), and thymol (2.3–18.9%) as the predominant constituents [15,17,19,26]. Similarly, investigations into wild Iranian populations of *S. boissieri* identified thirty-eight compounds, with carvacrol (70.1%) as the primary component, followed by γ-terpinene (6.8%), p-cymene (6.3%), and β-caryophyllene (4.0%) [14].

However, a notable difference from previously reported chemotypes is the very low thymol concentration (0.2%) in this sample [11]. Such variations in the carvacrol/p-cymene/γ-terpinene ratio—the biosynthetic precursors of phenolic monoterpenes—suggest that this sample belongs to a distinct carvacrol-type chemotype. As noted in some literature, these chemical fluctuations are typically influenced by environmental factors such as altitude, soil composition, or the specific phenological stage at the time of collection [15,27]. Ultimately, the high carvacrol content confirms the potent antimicrobial and antioxidant potential of this species for industrial applications.

### 2.2. LC-HRMS Analysis Results

To characterize the phenolic profile of the methanol extract of *Satureja boissieri*, analyses were performed using a Thermo Orbitrap Q-Exactive LC-HRMS system in full-scan mode with an ESI source and a library of thirty-two reference standards. Identification was achieved by comparing the MS data with published literature and authentic compounds. The major peaks were matched to available standards, and the method was validated for the compounds listed in Table 2. The specificity, linearity, precision, LOD, and LOQ of the LC-HRMS method and the uncertainty value of the measurement results are described in Table S1 in the supporting information.

In this study, a total of 27 phenolic constituents were quantified in the methanol extract of *Satureja boissieri* using a fully validated LC-HRMS method, supported by complete analytical validation for each constituent. While penduletin, (−)-epicatechin, (−)-epicatechin gallate, caffeic acid phenethyl ester, and verbascoside remained below the limit of detection (LOD), the remaining identified metabolites were successfully quantified (Table 2). This represents a comprehensive qualitative and quantitative profiling of *S. boissieri* with such an extensive range of constituents, distinguishing this research from previous qualitative reports on the genus.

The quantitative results demonstrate that the plant is an exceptionally rich source of bioactive metabolites, with a total phenolic content characterized by the following subgroup distributions: total flavonoids and derivatives: 10,589.14 mg/kg; total coumaric acids and derivatives: 48,419,32 mg/kg; total simple phenolics and others: 65,034.30 mg/kg. The chemical landscape of the extract is dominated by three primary constituents: syringic acid (56,647.96 mg/kg), rosmarinic acid (47,777.98 mg/kg), and hesperidin (6353.49 mg/kg), alongside a significant amount of fumaric acid (6218.96 mg/kg). Previous studies on this species showed that hesperidin and rosmarinic acid are determined as main compounds [18]. Rosmarinic acid, a polyphenolic compound, is recognized as a chemotaxonomic marker of the Lamiaceae family and has been identified as a predominant metabolite in most *Satureja* species analyzed to date [8,16,28,29]. A notable feature of the present study is the high concentration of syringic acid, which was found to be the most prominent phenolic constituent, exceedingly even the levels of rosmarinic acid. This finding is consistent with previous research conducted by our group [6,30]. Furthermore, while syringic acid has been reported as a major component in species such as *S. kitaibelii* and *S. montana* [31,32], Jerković et al. have suggested that syringyl derivatives, including syringic acid, can be considered reliable chemotaxonomic biomarkers for the characterization of *Satureja* taxa [33].

**Figure 1:**
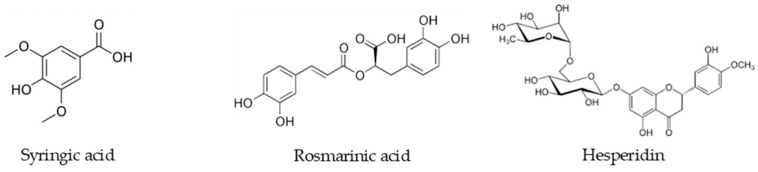
The structures of the most abundant phenolic compounds in methanol extracts of *S. boissieri*.

While rosmarinic acid is typically associated with the potent antioxidant and enzyme-inhibitory activities of the genus [8], the remarkable accumulation of syringic acid in *S. boissieri* suggests a unique metabolic profile that may synergistically contribute to its therapeutic potential, particularly in neuroprotection and oxidative stress mitigation [34]. Figure 1 depicts the chemical structures of the most abundant metabolites.

### 2.3. Reducing Ability Results

The electron-donating proficiency of the *S. boissieri* methanol extract was systematically evaluated using two of the most widely recognized spectrophotometric assays: the Ferric Reducing Antioxidant Power (FRAP) and the Cupric Reducing Antioxidant Capacity (CUPRAC). These methods assess the ability of the extract to reduce ferric (Fe^3+^) to ferrous (Fe^2+^) ions and cupric (Cu^2+^) to cuprous (Cu^1+^) ions, respectively. Reducing power reflects the extract’s ability to stabilize pro-oxidant metals, a key mechanism in preventing oxidative damage. The results, shown in Table 3, demonstrate a robust, concentration-dependent reducing capacity for the *S. boissieri* extract.

The extract showed strong ferric-reducing power with an absorbance of 0.505 at 700 nm and an excellent correlation coefficient (r2) of 0.9871. Although this value is lower than synthetic standards like BHA (2.347) and Trolox (2.119), it remains comparable to BHT (0.952) and α-tocopherol (0.957), demonstrating a significant ability to stop radical chain reactions.

Interestingly, the extract demonstrated superior performance in the CUPRAC assay, yielding an absorbance of 0.810 at 450 nm. Notably, this cupric-reducing capacity outperformed the natural standard α-tocopherol (0.693), positioning *S. boissieri* as a more potent natural alternative to traditional lipophilic and vitamin-based antioxidants for neutralizing metallic precursors of oxidative stress. This multifaceted reducing ability is likely a direct result of the high concentrations of syringic acid and rosmarinic acid identified in the LC-HRMS analysis, as these phenolics have multiple hydroxyl groups capable of donating electrons to stabilize metallic pro-oxidants.

### 2.4. Radical Scavenging Ability Results

The radical scavenging potential of the *S. boissieri* methanol extract was rigorously assessed using three distinct assays: DPPH***^•^***, ABTS***^•^***^+^, and DMPD***^•^***^+^. The results are expressed as IC_50_ values (µg/mL) in Table 4, with lower values indicating a higher antioxidant potency. The findings reveal that the extract exhibits broad-spectrum radical-scavenging capacity, particularly in the DPPH and ABTS systems. The extract exhibited an IC_50_ value of 11.74 µg/mL, which is remarkably close to the natural standard, α-tocopherol (11.31 µg/mL), and is significantly more effective than the synthetic antioxidant BHT (25.95 µg/mL). This high efficacy highlights the extract’s ability to donate hydrogen atoms to neutralize stable free radicals. In the ABTS assay, the extract showed an IC_50_ of 9.90 µg/mL. Although slightly higher than the standards (ranging from 5.07 to 8.37 µg/mL), this result confirms a strong capacity to scavenge cation radicals in both aqueous and organic phases. The extract demonstrated moderate activity in the DMPD assay (IC_50_ = 36.47 µg/mL). While the synthetic standards showed exceptionally low values in this specific assay, the extract’s consistent performance across all three radical types confirms its multifaceted antioxidant defense mechanism.

In a comprehensive study on *S. boissieri*, it was reported significant antioxidant activities in both ethanol and water extracts using DPPH, ABTS, FRAP, and CUPRAC assays [18]. The researchers identified hesperidin (5051 ± 247 ppb) and rosmarinic acid (4364 ± 214 ppb) as the primary phenolic constituents responsible for the biological activity. According to their findings, the ethanol extract exhibited potent radical scavenging with an IC_50_ values of 27.1 ± 1.3 µg/mL for DPPH and 22.7 ± 1.1 µg/mL for ABTS, while the reducing power measured by the CUPRAC method was found to be notably higher than that of the standard antioxidant ascorbic acid. In contrast to these findings, which identified hesperidin as the major compound in *S. boissieri*, our results highlight syringic acid as a primary metabolite, coinciding with a nearly two-fold increase in radical scavenging efficiency against DPPH radicals (IC_50_: 11.74 vs. 27.1 µg/mL).

Recent investigations into the antioxidant potential of the *Satureja* genus have revealed a high degree of efficacy across various species, consistently aligning with the profiles of standard antioxidants. In terms of radical scavenging, species such as *S. icarica*, *S. aintabensis*, and *S. spicigera* have demonstrated potent IC_50_ values for DPPH and ABTS radicals, typically ranging from 5.07 to 13.07 µg/mL [6,35,36,37]. These results are highly comparable to synthetic standards like BHA and α-tocopherol, although most species—including *S. aintabensis*—show notably lower efficiency in the DMPD assay than the standards [6]. In a separate study, the ABTS***^•^***^+^ scavenging capacity of *S. hortensis* essential oil and *S. montana* water extract was identified as the most potent among the tested extracts [38].

In terms of metal-reducing capabilities, it has been determined that methanol extracts of this genus generally exhibit superior performance compared to dichloromethane or hexane fractions. For example, the methanol extract of *S. pilosa* leaves reached a reduction capacity of 2.40 mmol Trolox/g, significantly exceeding that of the stem extracts [36]. Collectively, these findings reinforce the notion that *Satureja* species, particularly their polar extracts, are effective scavengers of reactive oxygen species and potent neutralizers of metallic pro-oxidants, thereby providing a solid reference point for the results of our study.

### 2.5. AChE and BChE Inhibition Results

Alzheimer’s disease (AD) is a complex neurodegenerative disorder driven by amyloid-beta accumulation, tau tangles, and oxidative stress. The detrimental role of reactive oxygen species (ROS) is central to AD pathogenesis, as oxidative stress promotes neuronal apoptosis and accelerates the formation of insoluble protein aggregates. This oxidative damage is intrinsically linked to the cholinergic hypothesis, which posits that the rapid hydrolysis of acetylcholine by AChE and BChE leads to cognitive decline. Therefore, identifying natural bioactive phenolics with dual functionality—mitigating oxidative stress while simultaneously inhibiting cholinesterase enzymes—represents a critical therapeutic strategy. The integration of such dual-action agents into the diet offers a promising approach to decelerate neurodegeneration by restoring cholinergic neurotransmission and neutralizing ROS-induced lesions [6,36,38,39,40].

Table 5 presents the inhibition percentages of acetyl and butyrylcholinesterase (AChE and BChE) enzymes, pertinent to AD, derived from methanol extracts and essential oils. Galantamine serves as the reference standard.

When the inhibitory effects of *S. boissieri* essential oil and methanol extract on AChE and BChE were evaluated, they exhibited a selective inhibition profile against acetylcholinesterase. It was determined that the methanol extract and essential oil had moderate AChE inhibition values of 35.3 ± 1.3% and 32.4 ± 3.3%, respectively. Although these values are lower than those of the potent synthetic standard galantamine (96.8 ± 1.3%), they are consistent with values reported in the literature for other species of the genus [6,30,36,37,40].

The inhibition of BChE by *S. boissieri* remained relatively low, particularly for the methanol (MeOH) extract (2.6 ± 3.8%), while the essential oil (EO) showed a more significant effect (20.4 ± 1.1%), outperforming the MeOH extract by a factor of nearly eight times. This divergence suggests that the bioactive constituents in the MeOH extract, specifically the high concentrations of syringic and rosmarinic acids, may have a higher binding affinity for the AChE enzyme pocket than for BChE. *S. boissieri* distinguishes itself by an AChE-selective mechanism that is of therapeutic interest for restoring cholinergic transmission in the early stages of Alzheimer’s disease without the systemic side effects of non-specific inhibition. The disparity in BChE inhibition is likely a result of the lipophilic nature of the EO’s primary constituents—carvacrol (45.2%) and p-cymene (26.0%)—which possess the necessary lipophilicity to penetrate the hydrophobic gorge of the BChE enzyme, a site less accessible to polar components [41]. This “hydrophobic preference” of BChE has been previously reported in other *Satureja* species where essential oils demonstrate superior BChE inhibition compared to their respective polar extracts [42]. Given that BChE becomes increasingly important in the progression of Alzheimer’s disease, the dual-enzyme inhibitory profile of *S. boissieri* EO underscores its potential as a multi-target neuroprotective agent.

## 3. Materials and Methods

### 3.1. Chemicals

DPPH***^•^***(2,2-diphenyl-1-picrylhydrazyl), BHA (butylated hydroxyanisole), ABTS***^•^***^+^ (2,2′-azino-bis(3-ethylbenzothiazoline-6-sulphonic acid), Trolox (6-hydroxy-2,5,7,8-tetramethylchroman-2-carboxylic acid), BHT (butylated hydroxytoluene), α-tocopherol ((2R)-2,5,7,8-tetramethyl-2-[(4R,8R)-4,8,12-trimethyltridecyl]-3,4-dihydro-2H-1-benzopyran-6-ol), neocuprine (2,9-dimethyl-1,10-phenanthroline), α-tocopherol were purchased from Sigma-Aldrich Chemie GmbH (Taufkirchen, Germany). were purchased commercially from Sigma-Aldrich GmbH, Steinheim, Germany. A list of the origin, brands, and purity of the chemicals and reference materials utilized in the study is as follows: Acacetin (>97% TRC, Toronto, ON, Canada), Apigenin (>97% TRC, Toronto, ON, Canada), Apigenin 7-glucoside (>97% EDQM CS, Strasbourg, France), Ascorbic acid (≥99% Sigma-Aldrich, St. Louis, MO, USA), Caffeic acid (≥98% Sigma-Aldrich, St. Louis, MO, USA), Caffeic Asit Phenethyl Ester (≥97% European pharmacopoeia reference standard, Strasbourg, France), Chlorogenic acid (≥97% European pharmacopoeia reference standard, Strasbourg, France), Chrysin (≥96% Sigma-Aldrich, St. Louis, MO, USA), Dihydrocapsaicin (≥97% Sigma-Aldrich, St. Louis, MO, USA), Dihydrokaempferol (>97% Phytolab, Vestenbergsgreuth, Germany), Fumaric acid (≥99% Sigma-Aldrich, St. Louis, MO, USA), Hesperidin (≥ 98% J&K, Beijing, China), Hispidulin (>97% TRC, Toronto, ON, Canada), Hyperoside (>97% TRC, Toronto, ON, Canada), Isosakuranetin (>97% Phytolab, Vestenbergsgreuth, Germany), Luteolin (95% Sigma-Aldrich, St. Louis, MO, USA), Luteolin 7-glucoside (>97% TRC, Toronto, ON, Canada), Luteolin-7-rutinoside (>97% Carbosynth limited, Compton, Berkshire, UK), Naringenin (≥95% Sigma-Aldrich, St. Louis, MO, USA), Naringin (≥90% Sigma-Aldrich, St. Louis, MO, USA), Nepetin (98% Supelco, Bellefonte, PA, USA), Orientin (>97% TRC Canada, Toronto, ON, Canada), Penduletin (>97% Phytolab Vestenbergsgreuth, Germany), Quercetin (≥95% Sigma-Aldrich, St. Louis, MO, USA), Quercitrin (>97% TRC, Toronto, ON, Canada), Rosmarinic acid (≥96% Sigma-Aldrich, St. Louis, MO, USA), Salicylic acid (≥98% Sigma-Aldrich, St. Louis, MO, USA), Syringic acid (≥95% Sigma-Aldrich, St. Louis, MO, USA), Vanillic acid (≥97% Sigma-Aldrich, St. Louis, MO, USA), Verbascoside (86.31% HWI Analytik GmbH, Rülzheim, Germany), (-) (-)-Epicatechin (≥90% Sigma-Aldrich, St. Louis, MO, USA), (-)-Epicatechin gallate (>97% TRC, Toronto, ON, Canada), (+)-trans taxifolin (>97% TRC, Toronto, ON, Canada).

### 3.2. Plant Material

**Figure 2:**
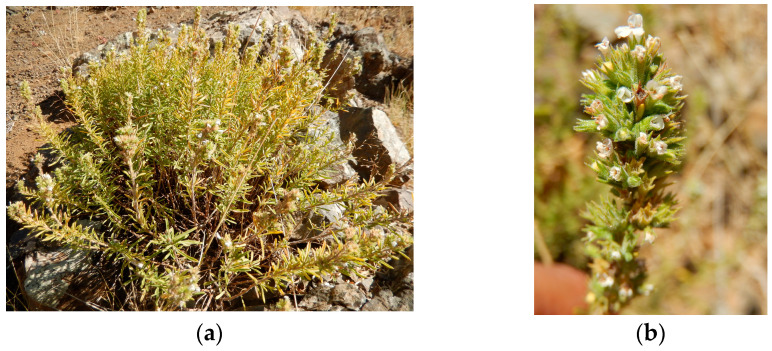
General appearance of *Satureja boissieri*: (**a**) its natural habitat on volcanic rocks in Adıyaman province (in situ); (**b**) detailed view of the aerial parts in flower.

*Satureja boissieri* was collected from Adıyaman province, Çelikhan, 8 km from Ulubaba Mountain to Yazıbaşı village, at 1700 m, on 09/02/2018, by Prof. Tuncay Dirmenci (Balıkesir University) and Prof. Dr. Turan Arabacı (Inonu University). It is grown on volcanic rocks and in surrounding steppe-like areas (Figure 2). The herbarium sample of this species was recorded and stored at Balıkesir University Necatibey Education Faculty Herbarium with the code TD 5207 (collector number Dirmenci 5207). The plant material was harvested from a representative population of multiple individuals to ensure genetic diversity. The analyzed aerial parts consisted of a mixture of leaves, flowers, and stems in their natural proportions observed during the collection period. The plant sample size (aerial parts) was determined to achieve a representative extract yield for multiple high-resolution analyses. To ensure the reliability of the findings, samples were collected from multiple individuals within the same population. This approach ensured that the resulting methanol extract reflected a homogenized, representative phytochemical profile of the species in its natural habitat, effectively accounting for individual biological variation.

### 3.3. Isolation of the Essential Oil

To obtain the essential oil of *S. boissieri*, 120 g of the dried aerial components were finely chopped and underwent a 4 h hydrodistillation process using a modified Clevenger-type system. The resulting distillate was captured in a 5 mL vial, while the interior surfaces of the apparatus were rinsed twice with 1 mL of diethyl ether using a glass Pasteur pipette to ensure maximum recovery. The collected oil was then dehydrated using anhydrous CaCl_2_ to remove any residual moisture. After the solvent was evaporated, the final yield (approximately 0.85 mL) was preserved at 4 °C to maintain its chemical integrity until the commencement of experimental analyses.

### 3.4. GC-MS Analysis

For the GC-MS analysis, essential oil of *S. boissieri* was injected into a Trace 1310 GC system coupled with a Thermo TSQ 9000 mass spectrometer (Thermo Scientific, Waltham, MA, USA). Chromatographic separation was achieved using a DB5-fused silica capillary column (60 m × 0.25 mm, ø, with a 0.5 mm film thickness), with helium as the carrier gas at a constant flow rate of 1 mL/min, following previously established protocols [40]. Mass spectra were recorded in electron ionization (EI) mode at 70 eV, scanning a range of *m*/*z* 50 to 650 amu.

Volatile components were identified by comparing their calculated Kovats Indices (relative to alkanes) and mass spectra (*m*/*z* 50–650) with authentic standards, literature data, and established spectral libraries. Technical details of GC-MS conditions and chromatographic parameters are provided in the Supporting Information.

### 3.5. Preparation of Plant Extracts

The extraction of phenolic compounds was performed using methanol, a common solvent widely recognized in Pharmacopeia-based protocols for extracting diverse phytochemicals from aromatic plants [43]. Air-dried and ground aerial parts of *S. boissieri* (10 g) were extracted by maceration in 100 mL of solvent using a 250 mL capped Schott flask. The process was maintained for four days with daily venting of the vessel. After this period, the mixture was filtered, and the solvent was removed under reduced pressure with a rotary evaporator.

### 3.6. LC-HRMS Analysis

The phenolic characterization of the extracts was executed using a high-resolution mass spectrometry (LC-HRMS), featuring a Thermo Orbitrap Q-Exactive system (Thermo Fisher Scientific, Waltham, MA, USA) integrated with a Troyasil C18 analytical column (150 × 3 mm, 5 μm; Istanbul, Türkiye). The analytical protocol was established by following a protocol whose validity had been demonstrated in our previous studies, to ensure the accuracy of metabolic profiling [6,35,36,44].

Method validation encompassed parameters such as specificity, accuracy, precision, and the determination of limits of detection (LOD) and quantification (LOQ). Data evaluation was conducted following EURACHEM/CITAC guidelines [45] and our prior reports, with comprehensive details on uncertainty assessment and validation metrics (linearity, precision, LOD, LOQ) provided in Table S1 of the Supporting Information.

Approximately 200 mg of the plant extract was weighed and added to a 5 mL volumetric flask. A measure of 3.5 mL of methanol was added, vortexed, placed in an ultrasonic bath at 24 °C, and kept until a clear mixture was obtained. A 200 μL of 1000 ppm dihydrocapsaicin solution used as an internal standard was added, and the final volume was completed with methanol. After being kept in the ultrasonic bath for 10 min, the solutions were kept at room temperature (24 ± 3 °C) in the dark for 10 min, filtered through a 0.45 μm Millipore Millex-HV filter, and each sample was placed in 1.5 mL vials, from which 2 μL of sample was injected into the LC-HRMS device for each run.

### 3.7. Antioxidant Activities

The antioxidant capacity of *S. boissieri* extract was evaluated through a multifaceted approach comprising three radical scavenging assays (DPPH, ABTS, and DMPD) and two metal-reducing potential tests (ferric (Fe^3+^) and cupric (Cu^2+^)) reducing power. All experimental assays were performed in triplicate (*n* = 3) to ensure reproducibility and statistical reliability, and the results were expressed as mean ± standard deviation (SD).

The DPPH (2,2-diphenyl-1-picrylhydrazyl) scavenging capacity was determined according to the Blois method [46] with minor modifications based on the spectrophotometric monitoring of the violet-colored stable radical’s bleaching at 517 nm in the presence of hydrogen-donating or electron-donating antioxidants. Similarly, the ABTS***^•^***^+^ (2,2-azino-bis-3-ethylbenzthiazoline-6-sulfonic acid) radical cation scavenging assay and the DMPD***^•^***^+^ (N,N-dimethyl-p-phenylenediamine) radical scavenging effect was employed to assess the samples’ decolorization capacity at 734 nm and 505 nm, respectively [47]. For all radical scavenging assays, butylated hydroxyanisole (BHA), butylated hydroxytoluene (BHT), Trolox and α-tocopherol were utilized as reference standards, with results expressed as IC_50_ values (µg/mL). The metal-reducing potential was first assessed using the Cupric Ion Reducing Antioxidant Capacity (CUPRAC) method, a colorimetric technique in which antioxidants reduce the neocuproine (Nc)-Cu^2+^ complex to a stable, colored Nc-Cu^+^ chelate with maximum absorbance at 450 nm. Additionally, the Ferric Ion (Fe^3+^) reducing ability was evaluated following the potassium ferricyanide procedure, where the reduction of ferric ions in the presence of phosphate buffer (pH 6.6) and potassium ferrocyanide leads to the formation of a Perl’s Prussian blue complex, measured at 700 nm after the addition of trichloroacetic acid and ferric chloride [6,35,36]. Collectively, these standardized methods provide a comprehensive profile of the extracts’ electron- and hydrogen-atom-transfer capabilities.

### 3.8. Anticholinergic Assays

To evaluate the anticholinergic potential of the extracts, acetylcholinesterase (AChE) and butyrylcholinesterase (BChE) inhibitory activities were determined using a spectrophotometric method based on the Ellman protocol [48], with minor modifications as previously detailed [6,36]. Acetylthiocholine iodide and butyrylcholine iodide were employed as substrates, while 5,5′-dithiobis(2-nitrobenzoic acid) (DTNB) was utilized to monitor anticholinesterase activity. Galantamine served as the positive control across all experiments, while a control representing 100% enzyme activity (without the inhibitor) served as the negative control. All experimental assays were performed in triplicate (*n* = 3) to ensure reproducibility and statistical reliability, and the results were expressed as mean ± standard deviation (SD). Comprehensive details regarding reagents, experimental conditions, and calculations were provided in our prior reports.

### 3.9. Statistical Analyses

Experimental results were obtained from triplicate measurements and expressed as the mean ± standard deviation (SD). To evaluate the statistical significance of the data, a one-way analysis of variance (ANOVA) was performed using the Statgraph Centurion XIX software package (Statgraphics Technologies, Inc., The Plains, VA, USA). Mean comparisons and the identification of distinct groups were determined using Duncan’s multiple-range test. In all evaluations, a *p*-value of less than 0.05 was considered statistically significant, and values below 0.01 were considered highly significant.

## 4. Conclusions

In this study, the chemical composition and multi-functional biological potential of the endemic *Satureja boissieri* were comprehensively evaluated. The essential oil (EO) was characterized as a distinct carvacrol-type chemotype (45.2%), while the methanol extract revealed an exceptionally rich phenolic profile dominated by syringic acid (56,647.96 mg/kg) and rosmarinic acid (47,777.98 mg/kg). A key highlight of this research is the identification of syringic acid as the primary metabolite, a finding that deviates from previous reports and suggests a unique metabolic adaptation of the species. Furthermore, intra-species variability, often driven by environmental factors, such as altitude and soil composition, may account for differences in secondary metabolite levels compared to earlier studies.

The biological assays demonstrated that *S. boissieri* possesses potent antioxidant and neuroprotective properties. The methanol extract exhibited broad-spectrum antioxidant defense, with radical-scavenging efficiencies (DPPH and ABTS) nearly comparable to standard antioxidants and a superior cupric-reducing capacity (CUPRAC) relative to α-tocopherol. Furthermore, the study revealed a significant divergence in cholinesterase inhibition: the polar extract showed an AChE-selective profile, whereas the essential oil demonstrated 8-fold greater potency against BChE (20.4%) compared to the methanol extract. This disparity underscores the “hydrophobic preference” of the BChE enzyme gorge, which is more effectively targeted by the lipophilic monoterpenes (carvacrol and p-cymene) found in the EO.

Overall, the dual-action capability of *S. boissieri*—mitigating oxidative stress while simultaneously modulating cholinergic enzymes—positions this endemic species as a promising multi-target candidate for the development of natural neuroprotective agents. These findings provide a strong scientific foundation for the potential industrial and pharmaceutical application of *S. boissieri* in the management of neurodegenerative disorders like Alzheimer’s disease. Future studies focusing on the in vivo efficacy and the synergistic effects of these identified major phenolics are recommended to further validate their therapeutic utility.

## Figures and Tables

**Table 1:** Essential oil composition of *Satureja* boissieri.

RI	RT	Compound	*Satureja boissieri*
Monoterpene Hydrocarbons			%
927	8.20	Tricyclene	t
930	9.31	α-Thujene	0.8
939	9.54	α-Pinene	0.5
954	10.06	Camphene	0.1
975	10.95	Sabinene	t
979	11.05	β-Pinene	0.1
991	11.59	β-Myrcene	1.3
1003	12.03	α-phellandrene	0.2
1009	12.24	3-Carene	t
1017	12.46	α-Terpinene	2.7
1025	12.88	p-Cymene	26.0
1029	12.95	Limonene	0.4
1060	13.93	γ-Terpinene	17.5
1096	14.87	Dimethylstyrene	0.2
Oxygenated monoterpenes			
1070	14.15	cis-Sabinene hydrate	0.1
1097	15.20	Linalool	0.6
1099	15.34	α-Pinene oxide	t
1141	15.86	cis-Verbenol	t
1143	16.45	cis-Sabinol	0.1
1145	16.63	Trans-verbenol	t
1159	16.78	β-Pinene oxide	t
1169	17.31	Borneol	0.1
1177	17.64	4-Terpineol	0.7
1183	17.87	p-Cymen-8-ol	0.1
1230	19.13	Nerol	t
1243	19.59	Carvone	0.2
1253	19.93	Geraniol	t
1373	23.07	Carvacrol acetate	0.1
Sesquiterpene hydrocarbons			
1377	23.28	α-Copaene	t
1380	23.53	β-Bourbonene	t
1421	24.42	β-Ylangene	0.6
1434	24.67	β-Gurjunene	t
1441	24.93	Aromadendrene	0.1
1500	25.84	α-Muurolene	t
1523	26.37	δ-Cadinene	0.1
1539	26.97	α-Cadinene	t
1546	27.45	α-Calacorene	0.3
Oxygenated sesquiterpenes			
1533	26.77	Nerolidol	t
1578	28.32	Spathulenol	0.3
1583	28.45	Caryophylenne oxide	0.3
Phenolics			
1290	20.75	Thymol	0.2
1291	20.97	p-Cymen-3-ol	0.4
1299	21.27	Carvacrol	45.2
1359	21.74	Eugenol	0.1
Other Hydrocarbons			
855	6.67	2-Hexanal	t
979	11.16	1-Octen-3-ol	0.2
991	11.73	3-Octanol	0.1
Total			99.7

RI, retention indices (calculated against n-alkanes); RT, the retention time (identification based on the retention times of compounds on the DB5 column; t, trace (<0.1%). GC-MS/FID analyses were replicated three times and SD of the measurements were found as <0.001).

**Table 2:** The quantity of phenolic compounds determined in chloroform (CHCl_3_) and methanol (MeOH) extracts of *S. boissieri* (mg/kg of extract) by LC/HRMS.

Compounds	MeOH	U % (k = 2) *	
Flavonoids and Derivatives			
Apigenin	87.97	11.54	
Chrysin	1.77	11.09	
Luteolin	355.26	12.41	
Luteolin-7-rutinoside	383.59	11.45	
Luteolin-7-glucoside	246.51	11.29	
Apigenin-7-glucoside	36.73	11.9	
Orientin	67.31	11.47	
Acacetin	203.97	11.36	
Hispidulin	68.18	11.23	
Nepetin	19.66	11.24	
Penduletin	<LOD **	11.81	
Quercetin	74.11	11.42	
Hyperoside	469.82	11.5	
Quercitrin	177.87	11.69	
- (−)-Epicatechin	<LOD **	11.91	
(−)-Epicatechin gallate	<LOD **	11.21	
(+)-t*rans* taxifolin	445.04	11.19	
Dihydrokaempferol	219.44	11.35	
Naringenin	1317.29	11.04	
Isosakuranetin	1.58	11.48	
Naringin	59.55	12	
Hesperidin	6353.49	11.15	
Coumaric acids and Derivatives			
Caffeic acid	349.17	11.07	
Chlorogenic acid	292.17	11.14	
Rosmarinic acid	47,777.98	11.63	
Caffeic acid phenethyl ester	<LOD **	<LOD **	11.38
Simple Phenolics and Others ***			
Syringic acid	56,647.96	12.37	
Salicylic acid	404.40	11.4	
Vanillic acid	724.77	11.61	
Verbascoside	<LOD **	12.08	
Ascorbic acid	1038.21	11.07	
Fumaric acid	6218.96	11.14	
	Total 124,042.76		

* U % (k = 2): Expanded measurement uncertainty calculated using a coverage factor of k = 2 at a 95% confidence level, accounting for method repeatability and laboratory precision; ** LOD: Limit of detection. *** Single-ring phenolics, organic acids, non-flavonoids.

**Table 3:** Reduction abilities of *Satureja boissieri* methanol extract and standard antioxidants for Fe^3+^ and Cu^2+^ at 30 μg/mL.

Antioxidants	Fe^3+^ Reducing		Cu^2+^ Reducing	
	λ (700 nm)	r^2^	λ (450 nm)	r^2^
BHA	2.347	0.9086	1.649	0.9584
BHT	0.952	0.9154	0.998	0.9834
Trolox	2.119	0.9586	1.108	0.9910
α-Tocopherol	0.957	0.9863	0.693	0.9934
*Satureja boissieri*	0.505	0.9871	0.810	0.9994

**Table 4:** IC_50_ (μg/mL) values for DPPH***^•^***, ABTS***^•^***^+^ and DMPD***^•^***^+^ scavenging activities of *Satureja boissieri* methanol extract and standard antioxidants.

Antioxidants	DPPH*^•^* Scavenging		ABTS*^•^*^+^ Scavenging		DMPD*^•^*^+^ Scavenging	
	IC_50_	r^2^	IC_50_	r^2^	IC_50_	r^2^
BHA	10.10	0.9015	5.07	0.9356	0.07	0.9465
BHT	25.95	0.9221	6.99	0.9350	0.07	0.9390
Trolox	7.05	0.9614	6.16	0.9692	0.072	0.9382
α-Tocopherol	11.31	0.9642	8.37	0.9015	-	-
*Satureja boissieri*	11.74	0.9559	9.90	0.9321	36.47	0.9646

**Table 5:** Acetylcholinesterase (AChE), and butyrylcholinesterase (BChE) enzymes’ inhibition (%) of the extract and essential oil of *S. boissieri* (40 µg/mL).

	AChE Inhibition (%)		BChE Inhibition (%)	
	Essential Oil	Methanol	Essential Oil	Methanol
*Satureja boissieri*	32.4 ± 3.3	35.3 ± 1.3	20.4 ± 1.1	2.6 ± 3.8
Galantamine	96.8 ± 1.3		83.3 ± 0.7	

## Data Availability

The original contributions presented in this study are included in the article/supplementary material. Further inquiries can be directed at the corresponding authors.

## Supplementary Materials

The following supporting information can be downloaded at: https://www.mdpi.com/article/10.3390/molecules31101710/s1, LC-HRMS Analysis [36,44,45,49,50,51,52,53], Table S1: Validation parameters and LC/MS-MS method developed for the secondary metabolites of the species; Figure S1: LC-HRMS chromatogram of *Satureja boissieri* (MeOH) extract; Figure S2: LC-HRMS chromatogram of internal standard (dihydrocapsaicin).

## Abbreviations

The following abbreviations are used in this manuscript:

GC-MS	Gas Chromatography–Mass Spectrometry
LC-HRMS	Liquid Chromatography–High-Resolution Mass Spectrometry
AD	Alzheimer’s disease
AChE	Acetylcholinesterase enzyme
BChE	Butyrylcholinesterase enzyme
BHA	Butylated hydroxyanisole
BHT	Butylated hydroxytoluene
DPPH	2,2-diphenyl-1-picrylhydrazyl
ABTS	2,2-azino-bis-3-ethylbenzthiazoline-6-sulfonic acid
DMPD	N,N-dimethyl-p-phenylenediamine
CUPRAC	Cupric Ions (Cu^2+^) Reducing Ability Assay
IC_50_	Half-maximal inhibitory concentration
