# Supplementary material for: Characterization of Phenolic and Essential Oil Constituents of Satureja boissieri Hausskn. ex Boiss and Evaluation of Antioxidant Potential
Source: Molecules. 2026 May 18;31(10):1710. doi: 10.3390/molecules31101710 (PMC13209572; doi:10.3390/molecules31101710)
Supplement: Supplementary file 1 [file molecules-31-01710-s001.zip › molecules-4261715-supplementary.pdf]

Supporting Information

Characterization of Phenolic and Essential Oil Constituents of *Satureja Boissieri* Hausskn. ex Boiss and Evaluation of Antioxidant Potential

Sema Çarıkçı [1,2,*], Tuncay Dirmenci [3], Ilhami Gulcin[4,5] and Ahmet C. Goren [6,7] *

[1] Vocational School, Izmir Demokrasi University, 35140 Izmir, Türkiye
[2] The Sustainable Environmental Studies Application and Research Centre, Izmir Demokrasi University, 35140 Izmir, Türkiye
[3] Department of Biology Education, Necatibey Faculty of Education, Balikesir University, 10145 Balikesir, Türkiye; dirmenci@balikesir.edu.tr
[4] Chemistry Department, Faculty of Science, Atatürk University, 25240 Erzurum, Türkiye; igulcin@atauni.edu.tr
[5] Rectorate of Agri Ibrahim Cecen University, 04100 Agrı, Türkiye
[6] Department of Chemistry, Faculty of Basic Sciences, Gebze Technical University, 41400 Kocaeli, Türkiye
[7] Troyasil HPLC Column Technologies, Doruk Analitik, Mehmet Akif Mah, Yumurcak Sok, No. 43, Istanbul 34744, Türkiye
* Correspondence: sema.carikci@idu.edu.tr (S.Ç.); acgoren@gtu.edu.tr (A.C.G.)

Table of Contents	Page
**S1.** Chemical Impurity	2
**S2.** LC-HRMS Analysis	3
**Table S1**: Validation parameters and LC/MS-MS method developed for the secondary metabolites of the species	5
**Figure S1.** LC-HRMS chromatogram of *Satureja boissieri* (MeOH) extract	6
**Figure S2.** LC-HRMS chromatogram of internal standard (dihydrocapsaicin)	8
**S3.** GC and GC-MS Analysis Conditions	

S1. *Chemical Impurity*

Ascorbic acid (≥99 % Sigma-Aldrich, St. Louis, MO, USA) , Chlorogenic acid, Fumaric acid (≥99 % Sigma-Aldrich, St. Louis, MO, USA), (-)-Epicatechin (≥90 % Sigma-Aldrich, St. Louis, MO, USA), (-)-Epicatechin gallate (>97% TRC, Toronto, ON, Canada), Verbascoside (86.31% HWI ANALYTIK GMBH), Orientin (>97% TRC, Toronto, ON, Canada), Caffeic acid (≥ 98 % Sigma-Aldrich, St. Louis, MO, USA , (+)-trans taxifolin (>97% TRC, Toronto, ON, Canada), Luteolin-7-rutinoside (>97% Carbosynth limited) , Vanilic acid (≥97 % Sigma-Aldrich, St. Louis, MO, USA), Naringin (≥90 % Sigma-Aldrich, St. Louis, MO, USA), Luteolin 7-glucoside (>97% TRC, Toronto, ON, Canada), Hesperidin (≥ 98% J&K, BEİJİNG, CHİNA), Rosmarinic acid (≥96 % Sigma-Aldrich, St. Louis, MO, USA), Hyperoside (>97% TRC, Toronto, ON, Canada), Dihydrokaempferol (>97% Phytolab, Vestenbergsgreuth, Germany), Apigenin 7-glucoside (>97% EDQM CS, Strasbourg, France), Quercitrin (>97% TRC, Toronto, ON, Canada), Quercetin (≥95% Sigma-Aldrich, St. Louis, MO, USA), Salicylic acid (≥98 % Sigma-Aldrich, St. Louis, MO, USA), Naringenin (≥95 % Sigma-Aldrich, St. Louis, MO, USA), Luteolin (95% Sigma-Aldrich, St. Louis, MO, USA), Nepetin (98% Supelco, Bellefonte, PA, USA), Apigenin (>97% TRC, Toronto, ON, Canada), Hispidulin (>97% TRC, Toronto, ON, Canada), Isosakuranetin (>97% Phytolab, Vestenbergsgreuth, Germany), Penduletin (>97% Phytolab, Vestenbergsgreuth, Germany), Caffeic Asit Phenethyl Ester (≥97% european pharmacopoeia reference standard), Chrysin (≥96% Sigma-Aldrich, St. Louis, MO, USA), Acacetin (>97% TRC, Toronto, ON, Canada), Syringic acid (≥95 % Sigma-Aldrich, St. Louis, MO, USA), Dihydrocapsaicin(≥ 97 % Sigma-Aldrich, St. Louis, MO, USA)

S2. LC-HRMS Analysis

S2.1. Sample Preparation for LC-HRMS

The LC-HRMS analysis was carried out as described in our previous studies [36,44,49-53]. Approximately 200 mg of the plant extract was weighed and added to a 5 mL volumetric flask. 3.5 mL of methanol was added, vortexed, placed in an ultrasonic bath at 24°C, and kept until a clear mixture was obtained. A 200 µL of 1000 ppm dihydrocapsaicin solution was used as an internal standard, and the final volume was completed with methanol. After being kept in the ultrasonic bath for 10 minutes, the solutions were kept at room temperature (24 ± 3°C) in the dark for 10 minutes, filtered through a 0.45 µm Millipore Millex-HV filter, and each sample was placed in 1.5 mL vials, from which 2 µL of sample was injected into the LC-HRMS device for each run.

S2.2. Standard Solutions

Standard solutions dissolved in methanol were prepared at 10 different concentrations (0.01, 0.05, 0.1, 0.3, 0.5, 1, 3, 5, 7, and 10 mg/L). A stock solution of 1000 mg/L dihydrocapsaicin (purity 97%) in methanol was used as an internal standard.

S2.3. LC-HRMS Conditions

Secondary metabolites of the *Satureja* boissieri were determined by using liquid chromatography-high-resolution mass spectrometry (LC-HRMS), which utilised an Orbitrap Q-Exactive mass spectrometer (Thermo Fisher Scientific Inc., Waltham, MA, USA) coupled with a Troyasil (Istanbul, Turkey) C18 column (150 x 3 mm, 5 µm particle size). In electrospray ionization (ESI) mode, a combination of 1% formic acid in water (mobile phase A) and 1% formic acid in methanol (mobile phase B) was used. A gradient program consisting of 90% A and 10% B for the first 60 s, 10% A and 90% B for the 7-14 min interval, and 90% A and 100% B for the 14-22 min interval was used. A mobile phase with a flow rate of 0.25 mL/min and a column at 25 °C were used. Environmental conditions were recorded as follows: room temperature 23.0 ± 3.0 °C and relative humidity (50 ± 15) % rh. The MS conditions used were as follows: sheath gas (Arb) 45, auxiliary gas (Arb) 10, positive ion voltage 3.50 kV, ion transfer tube temperature 300°C, and evaporator temperature 320°C. Identifications were made by comparing the retention times and target ions of the compounds in LC-ESI-HRMS [36,44,49-53].

S2.4. Method Validation

The method validation parameters used in this study were specificity, accuracy, precision, LOD and LOQ. The EURACHEM/CITAC guide and our previous studies were used to evaluate sources and quantify results [45]. Further information on the procedures for evaluating uncertainty can be found in the previous literature [36,44,49-53].

The uncertainty values for the measurement results are described in Table S1.

S2.4.1. Specificity

Specificity is the observation of only the analyte peak at the retention time of the target analyte in the presence of other components, such as impurities, matrix components, and degradation products. The target analyte is measured with high precision and accuracy and is identified in the matrix without interference. The specificity of the developed LC-ESI-HRMS method was determined

by direct analysis (blind) of the entire prepared different solvents, *Satureja* extract and added target analytes. The LC-HRMS method was selected in order to achieve the required selectivity and sensitivity in the matrix and to eliminate the negative effects of the interventions.

S2.4.2. Linearity, Accuracy, LOD and LOQ of the LCHRMS Method

In its simplest form, accuracy refers to how close the measurement is to the target reference value, i.e. the difference between the observations/measurements and the actual value. The percentage recovery value for each target analyte is one of the parameters controlled to ensure accuracy. This value was calculated from LC-ESI-HRMS data for each analyte according to the following formula:

Recovery % = Recovered concentration /injected concentration x 100

The recovery percentages for the studied compounds ranged from 81.55 to 101.91 percent.

Calibration curves based on analyte results from six replicate measurements using solutions of varying concentrations were used for the quantitative determination of secondary metabolites by LC-ESI-HRMS. The regression coefficient (R^2) and linear regression equation obtained from the determined curve are given in Table S1.

Limits of detection (LOD), limit of quantification (LOQ) of the method for every compound were determined using the following equation: LOD or LOQ = κSDa/b, where 3 for LOQ and κ= 3 for LOD.

S2.4.3. Measurement Uncertainty Assessment

The uncertainty parameters were determined as the uncertainties due to the purity of the standard, weighing, precision, and calibration curve for the applied method, and the uncertainty measurement was estimated using the GUM methodology in accordance with EURACHEM CITAC and ISO Guide 35 [45]. Detailed equations suitable for the detailed calculation method are given in our previous studies [36,44,49-53].

Table S1: Validation parameters and LC/MS-MS method developed for the secondary metabolites of the species

Compounds	Formula	m/z	Ionization mode	Linear range	Linear regression equation	LOD / LOQ	R^2	Recovery (%)
Ascorbic acid	$C_6H_8O_6$	175.0248	Negative	0.5-10	y=0.00347x-0.00137	0.39/1.29	0.999	96.2
Chlorogenic acid	$C_{16}H_{18}O_9$	353.0878	Negative	0.05-10	y=0.00817x+0.000163	0.02/0.06	0.999	96.68
Fumaric acid	$C_4H_4O_4$	115.0037	Negative	0.1-10	y=0.00061x-0.0000329	0.05/0.17	0.999	97.13
(-)-Epicatechin	$C_{15}H_{14}O_6$	289.0718	Negative	0.05-10	y=0.0172x+0.0002269	0.01/0.03	0.999	95.66
(-)-Epicatechin gallate	$C_{22}H_{18}O_{10}$	441.0827	Negative	0.05-10	y=0.00788x-0.0001875	0.01/0.03	1.000	96.54
Verbascoside	$C_{29}H_{36}O_{15}$	623.1981	Negative	0.1-10	y=0.00758x+0.000563	0.03/0.1	1.000	96.19
Orientin	$C_{21}H_{20}O_{11}$	447.0933	Negative	0.1-10	y=0.00757x+0.000347	0.01/0.03	0.999	96.22
Caffeic acid	$C_9H_8O_4$	179.0350	Negative	0.3-10	y=0.0304x+0.00366	0.08/0.27	0.999	94.51
(+)-*trans* taxifolin	$C_{15}H_{12}O_7$	303.0510	Negative	0.3-10	y=0.0289x+0.00537	0.01/0.03	0.998	91.66
Luteolin-7-rutinoside	$C_{27}H_{30}O_{15}$	593.1512	Negative	0.1-10	y=0.00879x+0.000739	0.01/0.03	0.999	93.05
Vanilic acid	$C_8H_8O_4$	167.0350	Negative	0.3-10	y=0.00133x+0.0003456	0.1/0.33	1.000	98.66
Naringin	$C_{27}H_{32}O_{14}$	579.1719	Negative	0.05-10	y=0.00576x-0.000284	0.01/0.03	0.999	101.91
Luteolin 7-glucoside	$C_{21}H_{20}O_{11}$	447.0933	Negative	0.1-7	y=0.0162x+0.00226	0.01/0.03	0.996	96.31
Hesperidin	$C_{28}H_{34}O_{15}$	609.1825	Negative	0.05-10	y=0.00423x+0.0000138	0.01/0.03	0.999	96.14
Syringic acid	$C_9H_{10}O_5$	197.0456	Negative	0.5-10	y=0.0000831x+0.000024	0.1/0.3	0.999	97.29
Rosmarinic acid	$C_{18}H_{16}O_8$	359.0772	Negative	0.05-10	y=0.00717x-0.0003067	0.01/0.03	0.999	99.85
Hyperoside	$C_{21}H_{20}O_{12}$	463.0882	Negative	0.05-10	y=0.0072x-0.00003096	0.01/0.03	1.000	96.62
Dihydrokaempferol	$C_{15}H_{12}O_6$	287.0561	Negative	0.3-7	y=0.0756x+0.0118	0.01/0.03	0.995	95.37
Apigenin 7-glucoside	$C_{21}H_{20}O_{10}$	431.0984	Negative	0.3-7	y=0.0246x+0.00306	0.01/0.03	0.996	96.07
Quercitrin	$C_{21}H_{20}O_{11}$	447.0933	Negative	0.05-10	y=0.0179+0.0003331	0.01/0.03	0.999	97.0
Quercetin	$C_{15}H_{10}O_7$	301.0354	Negative	0.1-10	y=0.0509x+0.00467	0.01/0.03	0.998	96.41
Salicylic acid	$C_7H_6O_3$	137.0244	Negative	0.3-10	y=0.0361x+0.00245	0.01/0.03	0.998	92.88
Naringenin	$C_{15}H_{12}O_5$	271.0612	Negative	0.1-10	y=0.0281x+0.00182	0.01/0.03	1.000	86.65
Luteolin	$C_{15}H_{10}O_6$	285.0405	Negative	0.1-10	y=0.117x+0.00848	0.01/0.03	0.998	96.68
Nepetin	$C_{16}H_{12}O7$	315.0510	Negative	0.05-10	y=0.0853x+0.00269	0.01/0.03	0.999	97.76
Apigenin	$C_{15}H_{10}O_5$	269.0456	Negative	0.3-10	y=0.104x+0.0199	0.01/0.03	1.000	81.55
Hispidulin	$C_{16}H_{12}O_6$	301.0707	Pozitif	0.05-10	y=0.02614x+0.0003114	0.01/0.03	0.999	98.36
Isosakuranetin	$C_{16}H_{14}O_5$	285.0769	Negative	0.05-10	y=0.0235x+0.000561	0.01/0.03	0.999	96.56
Penduletin	$C_{18}H_{16}O_7$	343.0823	Negative	0.3-10	y=0.0258x+0.00253	0.01/0.03	0.999	83.43
Caffeic asit phenethyl ester	$C_{17}H_{16}O_4$	283.0976	Negative	0.3-7	y=0.255x+0.0477	0.01/0.03	0.996	94.42
Chrysin	$C_{15}H_{10}O_4$	253.0506	Negative	0.05-7	y=0.0964x-0.0002622	0.01/0.03	0.999	87.92
Acacetin	$C_{16}H_{12}O_5$	283.0612	Negative	0.05-7	y=0.046x+0.0001875	0.01/0.03	1.000	87.52

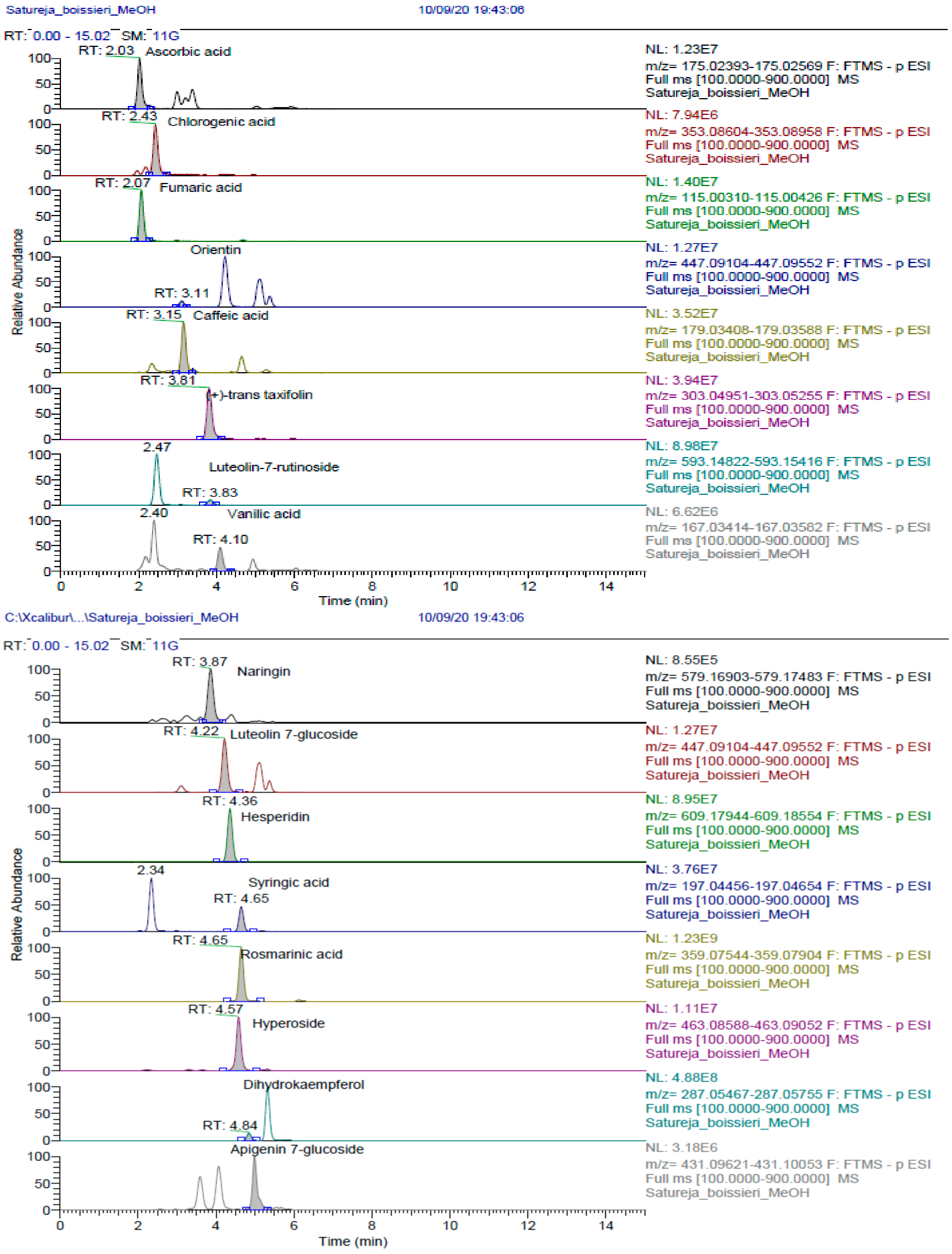

Figure S1. LC-HRMS chromatogram of *Satureja boissieri* (MeOH) extract

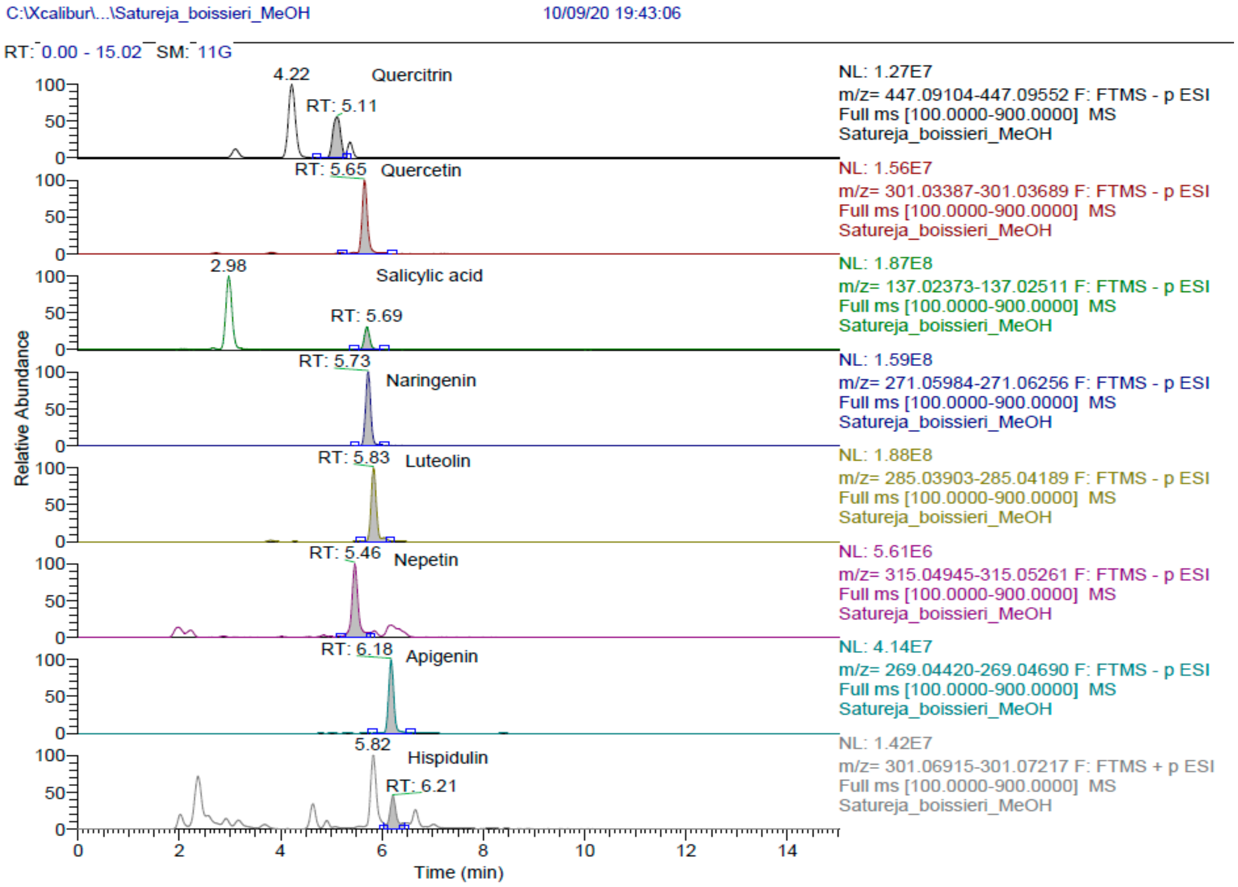

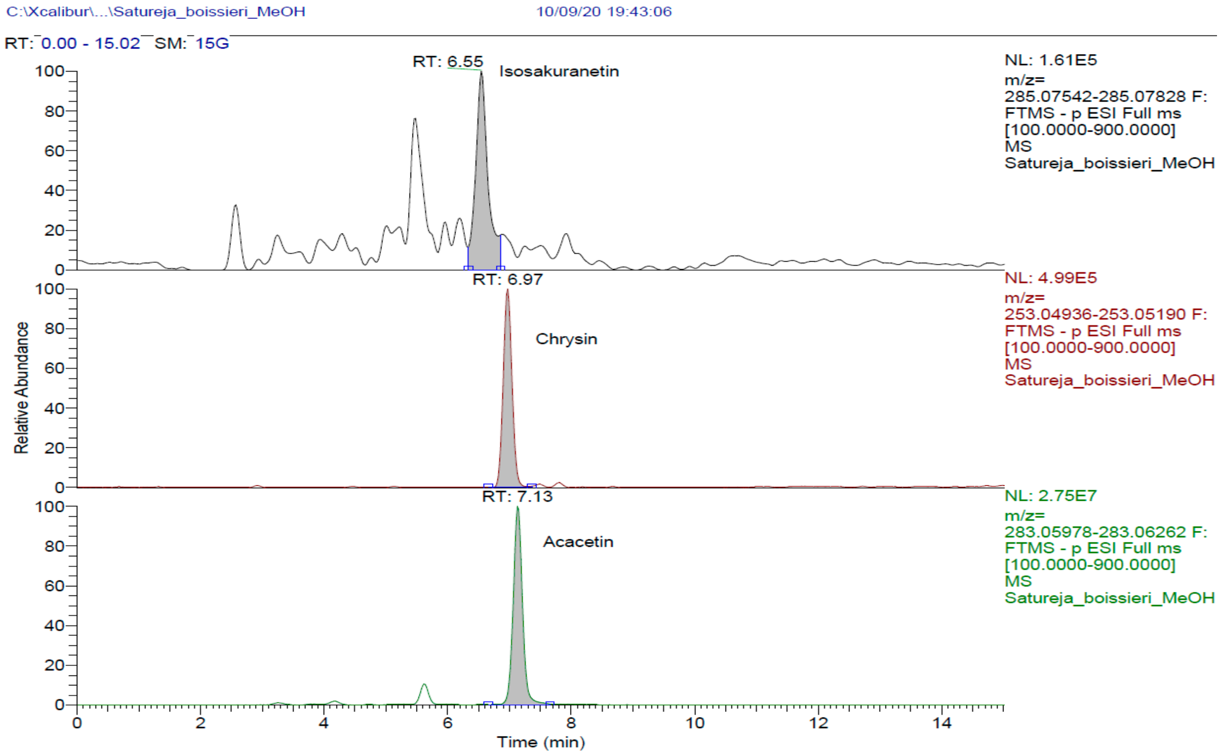

Figure S1. Continued

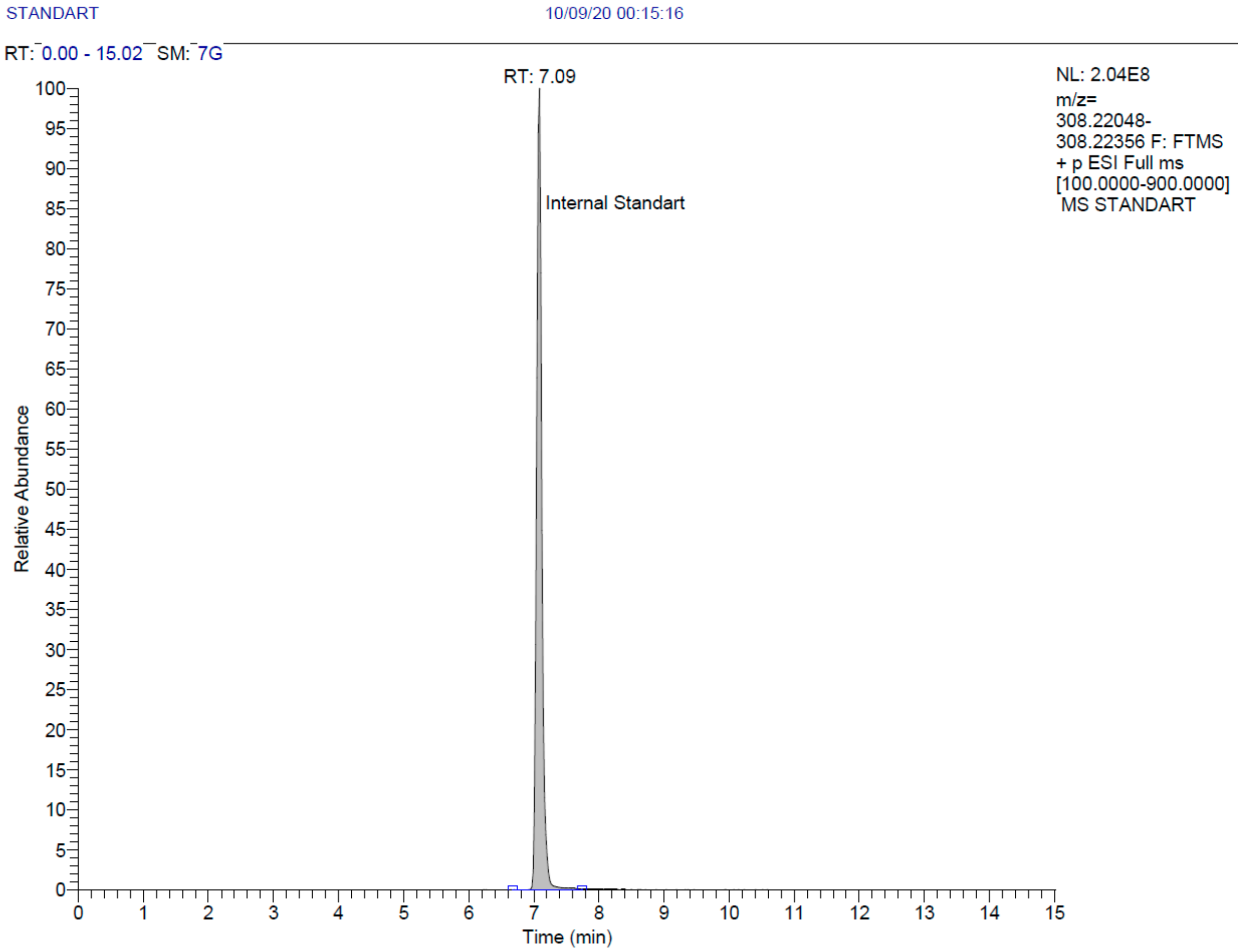

Figure S2. LC-HRMS chromatogram of internal standard (dihydrocapsaicin**)**

S3. GC and GC-MS Analysis Conditions

The chemical characterization of the essential oil (EO) was performed using capillary gas chromatography (GC) and gas chromatography-mass spectrometry (GC-MS) platforms.

S.3.1. GC-MS and GC-FID Parameters

For the GC-MS analysis, the EO was diluted in CH_2Cl_2 (1:10) and injected into a Trace 1310 GC system coupled with a Thermo TSQ 9000 mass spectrometer (Thermo Scientific, Waltham, MA, USA). Chromatographic separation was achieved using a DB5-fused silica capillary column (60 m × 0.25 mm, ø, with a 0.5 mm film thickness), with helium as the carrier gas at a constant flow rate of 1 mL/min. The oven temperature program was initiated at 100°C for 5 min, increased to 240°C at a gradient of 4°C/min, and maintained at the final temperature for an additional 5 min. The injector, ion source, and MS interface temperatures were set at 250°C, 220°C, and 240°C, respectively. Samples (0.5 μL) were injected in triplicate using a 1:20 split ratio. Mass spectra were recorded in electron ionization (EI) mode at 70 eV, scanning a range of *m/z* 50 to 650 amu. Simultaneous GC-FID analysis was executed on a Trace 1310 system under identical chromatographic conditions to ensure consistent elution patterns, with the FID temperature maintained at 300°C.

S.3.2. Compound Identification

The identification of volatile constituents was primarily based on the calculation of Kovats Indices (KI) relative to a homologous series of *n*-alkanes. The obtained mass spectra and retention times were rigorously compared with authentic standards, literature data, and established spectral libraries (NIST, Wiley, and the ILMER library of Bezmialem Vakif University). To verify the library matches, an Ultra Kit WRK 105 terpene mixture was used for co-injection. Furthermore, the identities of major components, including thymol, carvacrol, spathulenol, and caryophyllene oxide, were confirmed using previously isolated authentic standards.

References

36. Kınoğlu, B.K.; Gülçin, İ.; Gören, A.C. Quantification of Secondary Metabolites of *Satureja pilosa* (Lamiaceae) by LC-HRMS and Evaluation of Antioxidant and Cholinergic Activities. *Rec. Nat. Prod.* **2024**, *18*, 674-686. **http://doi.org/10.25135/rnp.489.2410.3378**
44. Özer, Z.; Çarıkçı, S.; Kılıç, T.; Selvi, S.; Gören, A.C. Determination of the effect of different drying methods on secondary metabolites of *Lavandula pedunculata* (Mill.) Cav. subsp. *cariensis* (Boiss.) Upson & S. Andrews by LC-HRMS. *J. Chem. Metrol.* **2024**, *18*(2), 124-133. **http://doi.org/10.25135/jcm.119.2411.3382**
45. EURACHEM/CITAC. Quantifying Uncertainty in Analytical Measurements, 2nd ed.; Guide CG4; Eurachem: Teddington, UK, 2000.
49. Dikici, E.; Altın, S.; Alp, C.; Işık, M.; Köksal, E.; Gülçin, İ. Determination of secondary metabolites of *Cydonia oblonga* (Quince) by LC-MS/MS method together with evaluation of its antioxidant and cholinergic potentials. *J. Chem. Metrol.* **2024**, *18*, 146-164. **http://doi.org/10.25135/jcm.120.2411.3380**
50. Çarıkçı, S.; Kılıç, T.; Dirmenci, T.; Gören, A.C. Phenolic compounds from section Majorana (Mill.) Benth of *Origanum* L. species extracts via validated LC-MS/MS method. *J. Chem. Metrol.* **2022**, *16*, 147-151. **http://doi.org/10.25135/jcm.80.2211.2667**
51. Mutlu, M.; Bingöl, Z.; Uç, E.M.; Köksal, E.; Gören, A.C.; Alwasel, S.H.; Gülçin, İ. Comprehensive metabolite profiling of cinnamon (*Cinnamomum zeylanicum*) leaf oil using LC-HR/MS, GC/MS, and GC-FID: determination of antiglaucoma, antioxidant, anticholinergic, and antidiabetic profiles. *Life*, **2023**, *13(1)*, 136. **https://doi.org/10.3390/life13010136**
52. Karageçili, H.; Polat, T.; Yılmaz, M. A.; Fidan, M.; Karaismailoğlu, M. C.; Gülçin, İ. Evaluation of the antioxidant, Antidiabetic and Anti-Alzheimer Effects of *Capsella bursa-pastoris*-Polyphenolic profiling by LC-MS/MS. *Rec. Nat. Prod.* 2024, *18*(*6*), 643 - 662. **http://doi.org/10.25135/rnp.489.2410.3353**
53. Yazıcı-Tütüniş, S.; Alim Toraman, G.Ö.; Dincel, E.D.; Tufan, S.; Akalın, E.; Tan, E.; Guzeldemirci-Ulusoy, N.; Goren A.C.; Miski, M.; Topçu, G. Tan, N. In Silico and in Vitro Antibacterial Evaluation of Eight Anatolian *Salvia* Species with Chemical Profiling by LC-HRMS. *Sci. Rep.* **2025**, *15*(1), 31404. **https://doi.org/10.1038/s41598-025-15803-1**
